# Novel *NOG* (p.P42S) mutation causes proximal symphalangism in a four-generation Chinese family

**DOI:** 10.1186/s12881-019-0864-1

**Published:** 2019-08-01

**Authors:** Yanwei Sha, Ding Ma, Ning Zhang, Xiaoli Wei, Wensheng Liu, Xiong Wang

**Affiliations:** 1 Department of Reproductive Medicine, Xiamen Maternity and Child Care Hospital, Xiamen, 361005 Fujian China; 2 Reproductive Medicine Center, Affiliated Yantai Yuhuangding Hospital of Qingdao University, Yantai, 264000 Shandong China; 3 School of Pharmaceutical Sciences, Xiamen University, Xiamen, 361005 Fujian China

**Keywords:** Proximal symphalangism, Whole genome sequencing, Missense mutation, NOG

## Abstract

**Background:**

Proximal symphalangism (SYM1; OMIM 185800), also called Cushing’s symphalangism, is an infrequent autosomal dominant disease. An SYM1 patient typically features variable fusion of proximal interphalangeal joints in the hands and feet.

**Methods:**

We recruited a four-generation Chinese non-consanguineous family with SYM1. We examined their hands and feet using X-rays to confirm fusion of proximal interphalangeal joints. We evaluated their audiology using standard audiometric procedures and equipment. Then, we identified genetic variants using whole exome sequencing and validated mutations using Sanger sequencing. Mutation pathogenicity was analyzed with bioinformatics.

**Results:**

Radiographs revealed proximal-joint fusion of fingers and toes in the patients. Two elderly individuals (II:1 and II:4) exhibited slight hearing loss. Additionally, we detected a novel heterozygous missense mutation in exon 1 of *NOG* (NM_005450) c.124C > T, p.(Pro42Ser) in all patients. This c.124C > T mutation is highly conserved across multiple species and the p.(Pro42Ser) variation is potentially highly pathogenic.

**Conclusion:**

Our results suggest that heterozygous c.124C > T, p.(Pro42Ser) in *NOG* is a novel mutation that causes human SYM1 phenotype.

**Electronic supplementary material:**

The online version of this article (10.1186/s12881-019-0864-1) contains supplementary material, which is available to authorized users.

## Background

Proximal symphalangism (SYM1; MIM# 185800) is a rare condition characterized by ankylosis of proximal interphalangeal joints (PIP, those between the first (also called proximal) and second (intermediate) phalanges) in fingers and toes, due to carpal and tarsal bone fusion [1]. In some affected individuals, fusions are concomitant with conductive hearing loss [2–5], hypermetropia [6–8], deformed facies [7], absence of digit flexion creases [1, 9], curtate metacarpals, and semideveloped distal phalanges [7, 10–12].

SYM1 appears to be an autosomal dominant disorder with prominent familial inheritance characteristics [13–15]. Two potential pathogenic genes have been identified in SYM1 patients: noggin (*NOG*, MIM# 602991) and *GDF5* (MIM# 601146). *NOG* mutations are the main cause of SYM1, accounting for most case reports of this disease.

Here, we investigated a four-generation non-consanguineous Chinese family with SYM1. Whole exome sequencing revealed a novel heterozygous missense mutation in *NOG*. Our findings expanded current understanding of the relationship between *NOG* mutations and SYM1. Overall, this work provides more insight on SYM1 for researchers and clinicians.

## Methods

### Subjects

**Fig. 1:**
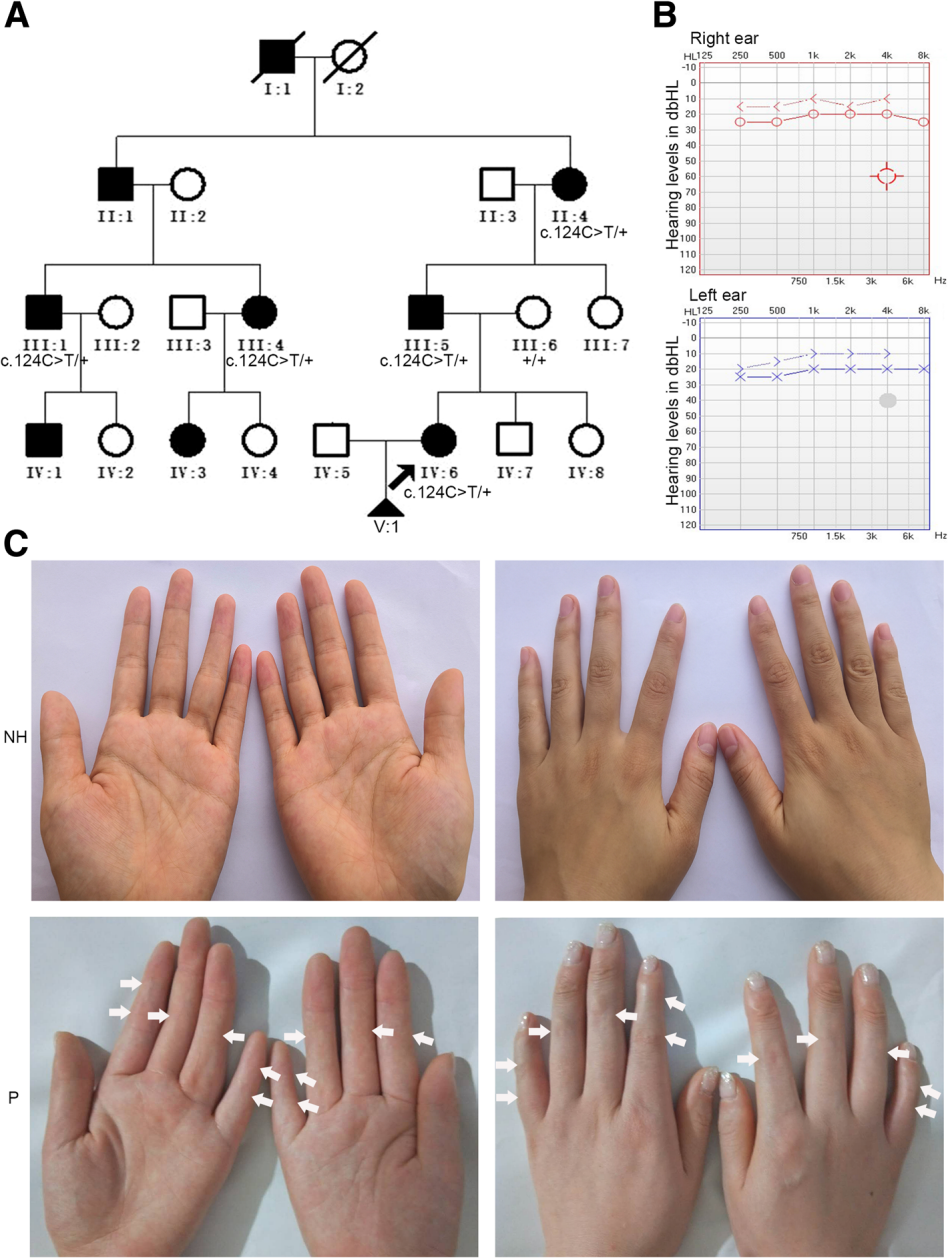
A four-generation Chinese family with autosomal dominant SYM1. **a** Black squares or circles represent individuals suffering from SYM1. The black arrow indicates the proband (IV: 6). Individuals I:1 has the abnormal phenotype, according to the descriptions of their relatives. **b** Audiogram from the proband showing normal hearing level. **c** Photographs showing the proband’s hand (The places where the curved creases disappear are marked with white arrows) and the hand of a normal control

A non-consanguineous Chinese family with SYM1 was recruited from Shandong province. The family comprised 21 individuals across four generations, with nine individuals affected. Diagnostic traits were as follows: proximal symphalangism (SYM1), variable fusion of PIP joints in hands and feet; multiple synostosis syndrome (SYNS), multiple clinical features that include facial dysmorphism, progressive fusion of PIP joints, fusion of multiple other joints (including spine) at variable degrees, and conductive hearing loss; temtamy preaxial brachydactyly syndrome (TPBS), limb malformations, short stature, and hearing loss. The proband (IV:6, Fig. 1) suffered from congenital PIP joint stiffness in fingers and toes. The proband was pregnant naturally 4 year ago; at 7 months, disease-induced abnormal fetal development resulted in the need for induced labor. Pedigree and family disease history were recorded in detail. Whole peripheral blood samples of six family members (II:4, III:1, III:4, III:5, III:6, and IV:6 in Fig. 1a) were obtained.

All participants signed a written informed consent form. The study was approved by the Ethics Committee of Affiliated Yantai Yuhuangding Hospital of Qingdao University.

### Whole-exome sequencing (WES) and bioinformatics analysis

WES of blood samples was performed as described previously [16]. The proband genomic DNA was isolated for exome enrichment using the TruSeq Exome Enrichment kit (Illumina, San Diego, CA, USA), following manufacturer protocol. The proband exome was sequenced in Illumina Hiseq 2000, then was analyzed and annotated following an in-house pipeline. Briefly, low-quality reads or adapters were first removed before cleaned reads were mapped to the human reference genome (GRCh37) in Burrows-Wheelers Aligner. The resultant bam file was sorted using SAMtools. Only high-quality alignments were used for the remaining analyses to guarantee variant-calling accuracy. Picard was employed to mark duplicates from PCR amplification. Single nucleotide variants (SNVs) and indels were called in SAMtools, while CoNIFER was applied to detect copy number variations (CNVs). Subsequently, SNVs, indels, and CNVs were annotated in ANNOVAR with several prediction tools. Allele frequency was obtained after comparing each variant against multiple public databases, including Single Nucleotide Polymorphism, 1000 Genomes Project, NHLBI Exome Sequencing Project (ESP) 6500, and Exome Aggregation Consortium. In terms of possible influence on protein function, nonsense, frame-shift, splice-site variants, or missense variants were evaluated using prediction tools such as Sorting Intolerant from Tolerant (SIFT), Polymorphism Phenotyping version 2 (PolyPhen-2) and MutationTaster.

#### Prioritization of candidate variants and disease-associated genes

Based on variant annotations, a series of prioritization strategies were applied to identify candidate variants associated with disease phenotypes. First, variants in repeats or segmental duplications were excluded. Other variants excluded included those outside exonic and splicing regions, those with minor allele frequency (MAF) > 0.01 according to public databases, synonymous variants, and non-conservative variants (score ≤ 2 according to GERP++). Based on predictions from SIFT (D), Polyphen2 (D), and MutationTaster (D), variants that do not damage protein function were also excluded. The remaining variants and related genes were considered disease-causing candidates. They were then ranked by Phenolyzer, a tool using prior information to implicate genes involved in diseases. The inputted phenotype term was “symphalangism.”

### Validation of mutations

Sanger sequencing was used to validate *NOG* mutations in the proband (IV:6, Fig. 1a) and her family (II:4, III:1, III:4, III:5, III:6 and IV:6 in Fig. 1a). The following primers were used: forward, GAGGGAAGTGCCCCTAGAAC and reverse, ATGAAGCCTGGGTCGTAGTG.

Noggin amino-acid sequences from different species were compared using the Cobalt tool. Wild-type and mutant noggin protein structure was modeled by SWISS-MODEL [17], based on the 1M4U file from PDB [18]. Three-dimensional images were built in UCSF Chimera following their operation manual [19].

## Results

### A four-generation Chinese family with SYM1

The proband (IV:6) and her four-generation non-consanguineous family were recruited for this study. Pedigree analysis showed an autosomal dominant genetic type (Fig. 1a). The nine affected individuals ranged from 20 to 80 years old.

**Fig. 2:**
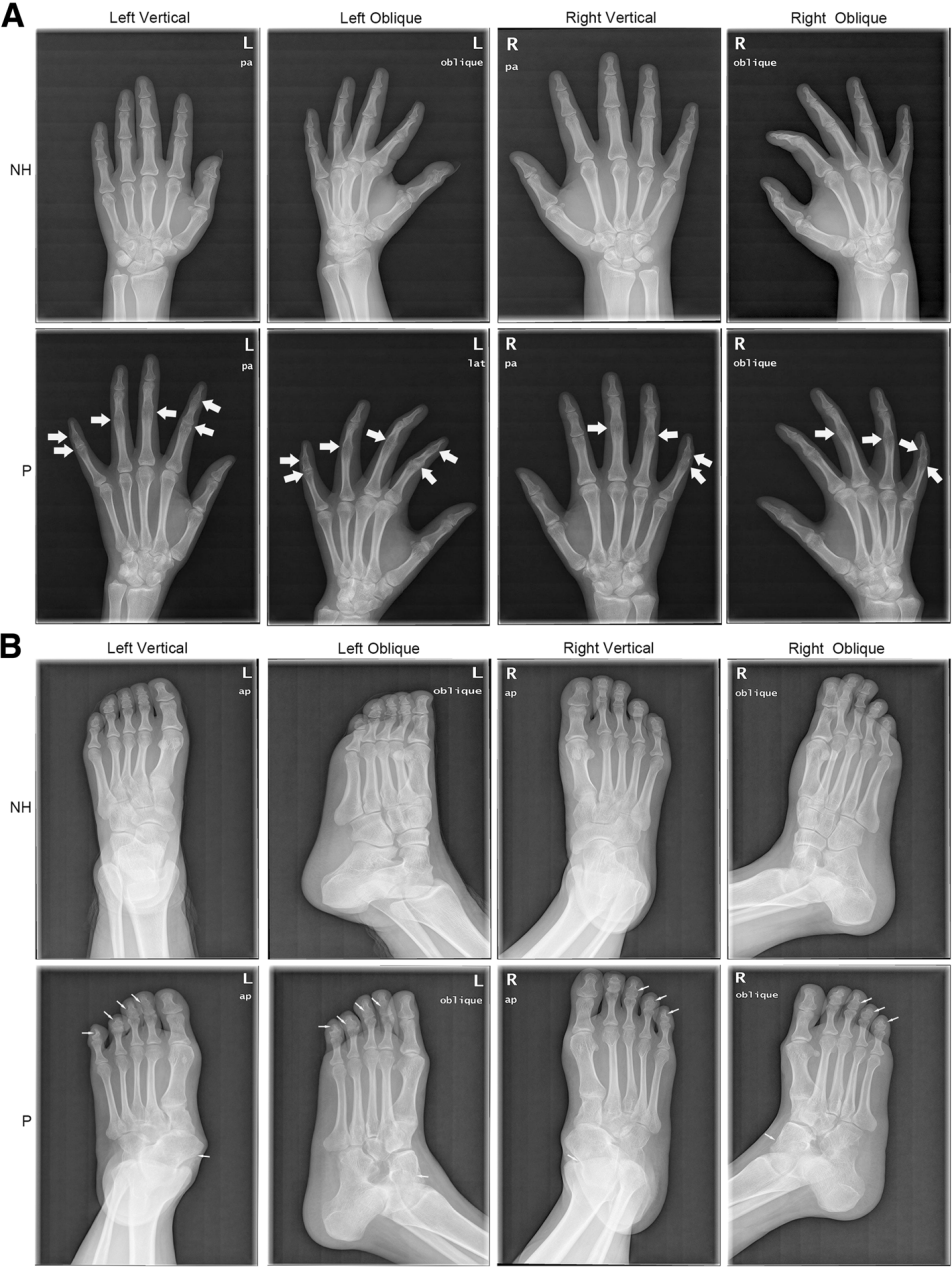
Radiographs of hands and feet of the proband and her normal mother. **a** Vertical and oblique views of both hands are shown. Arrows indicate interphalangeal-joint fusion in the digits. **b** Vertical and oblique views of both feet are shown. Arrows indicate interphalangeal-joint fusion in the digits and talonavicular synostosis

The proband had normal facial features. Audiologic evaluation showed that her hearing level was normal hearing level (Fig. 1b), indicating the lack of additional rigidity in the proband’s auditory ossicles. Her fingers are straight and some have no curved creases (Fig. 1c); she cannot form a perfect fist. Vertical and oblique radiographs of her fingers and toes revealed conspicuous fusions of PIP joints (Fig. 2). Specifically, all fingers except the forefingers had conspicuous fusions (Fig. 2a), while the feet displayed fused distal interphalangeal joints and talonavicular synostosis (Fig. 2b). Additionally, she did not present thumb enlargement or hyperopia. Clinical examination revealed similar characteristics to the proband in her family members who participated; all were diagnosed with SYM1 (Additional file 1). None suffered hearing loss except for two elderly individuals (II:1 and II:4; Additional file 1).

### Heterozygous *NOG* mutation identified in patients

The majority of patients with SYM1 are caused by genetic mutations. To identify the origin of chromosomal base mutations, genomic DNA was extracted from the whole blood of each SYM1 patient. The genomic DNA of the proband was subjected to WES analysis and all the variants observed were summarized in Additional file 2.

**Fig. 3:**
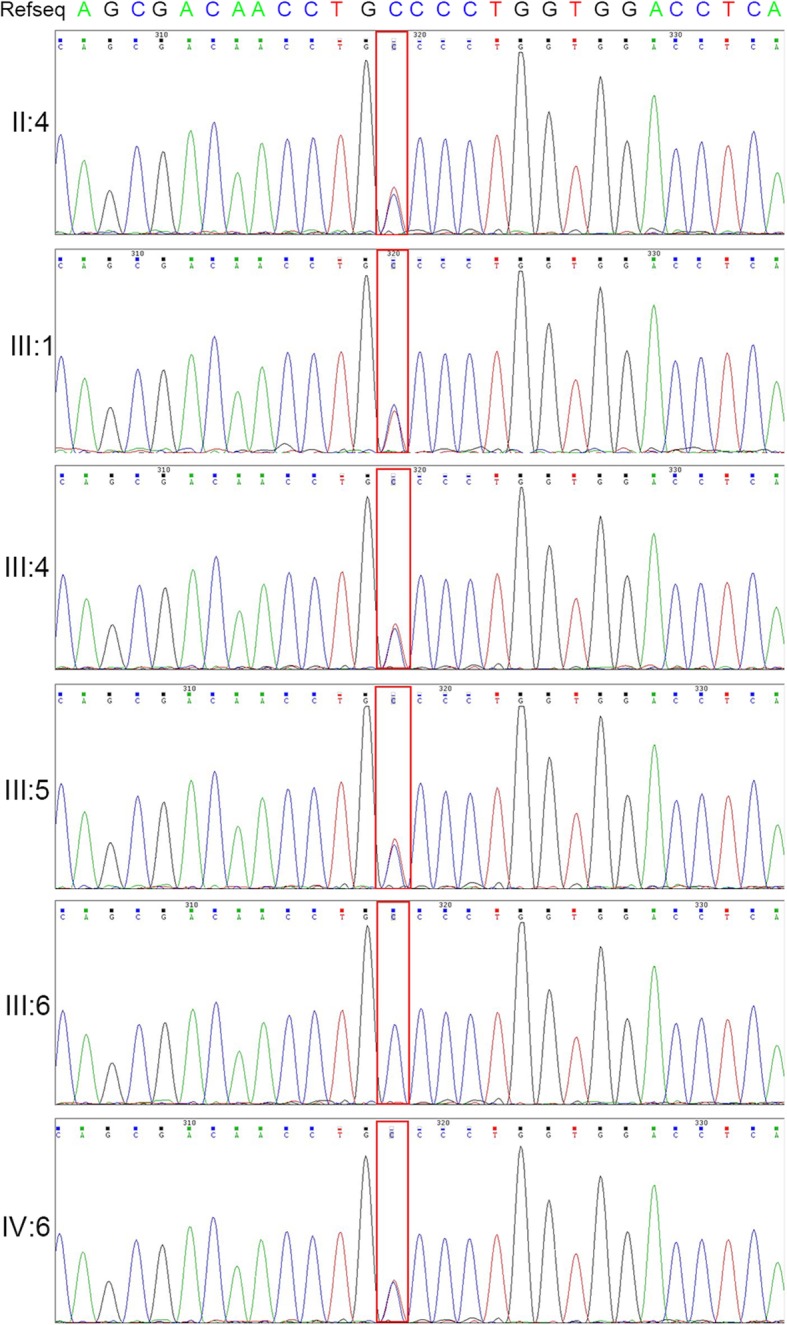
Identification of a novel *NOG* mutation. DNA sequencing profile around the position c.C124. The proband’s mother (III: 6) is a normal individual without SYM1. The red rectangle indicates the heterozygous mutation in the proband (IV: 6) and her affected family (II: 4, III:1, III:4, III:5)

After exclusion of irrelevant or meaningless mutations (detailed in Methods), we identified a novel heterozygous *NOG* variation c.124C > T, p.(Pro42Ser) in the proband (Fig. 3a, line 6). Validation with Sanger sequencing was performed for six members of the family (II:4, III:1, III:4, III:5, III:6 and IV:6 in Fig. 1a). This mutation was also present in the other patients with SYM1 (Fig. 3a, line1-line4), but not in the proband’s unaffected mother (Fig. 3a, line 5). These results suggested that SYM1 is an autosomal dominant disease.

### Effect of the c.124C > T, p.(Pro42Ser) mutation

We identified c.124C > T, p.(Pro42Ser) in *NOG* exon of patients with SYM1. Encoded amino acid by this mutation is in the functional domain of noggin. Bioinformatics analysis further indicated that the mutation may alter protein function and could be highly pathogenic (Table 1). Assessment of mutation frequency in the general population suggested that it is rare (Table 1). Taken together, the location, pathogenicity, and rarity of the novel *NOG* mutation suggest that it is the main cause of SYM1 among our sample.

**Table 1:** In silico analysis of *NOG* mutations

Mutation	Amino acid change	Polyphen-2^a^	SIFT^b^	Mutation Taster^c^	SNPs& GO^d^	ExAC (total)^e^	ExAC_EA^f^	1000G_ALL^g^	1000G_EA^h^
c.C124T	p.Pro42Ser	Probably damaging (1)	Deleterious (0.004)	Disease causing (1)	Disease (0.990)	0	0	0	0

^a^Polyphen-2 (http://genetics.bwh.harvard.edu/pph2/). Prediction Scores range from 0 to 1 with high scores indicating probably or possibly damaging

^b^SIFT, that is, Sorting Intolerant From Tolerant (http://sift.jcvi.org/). Scores vary between 0 and 1. Variants with scores close or equal to 0 are predicted to be damaging

^c^Mutation Taster (http://www.mutationtaster.org/). The probability value is the probability of the prediction, that is, a value close to 1 indicates a high “security” of the prediction

^d^SNPs & GO (http://snps.biofold.org/snps-and-go/). Probability:disease probability (if > 0.5 mutation is predicted disease)

^e^Frequency of variation in total of ExAC database

^f^Frequency of variation in East Asian population of ExAC database

^g^Frequency of variation in total of 1000 Genomes database (A Deep Catalog of Human Genetic Variation)

^h^Frequency of variation in East Asian population of 1000 Genomes database

**Fig. 4:**
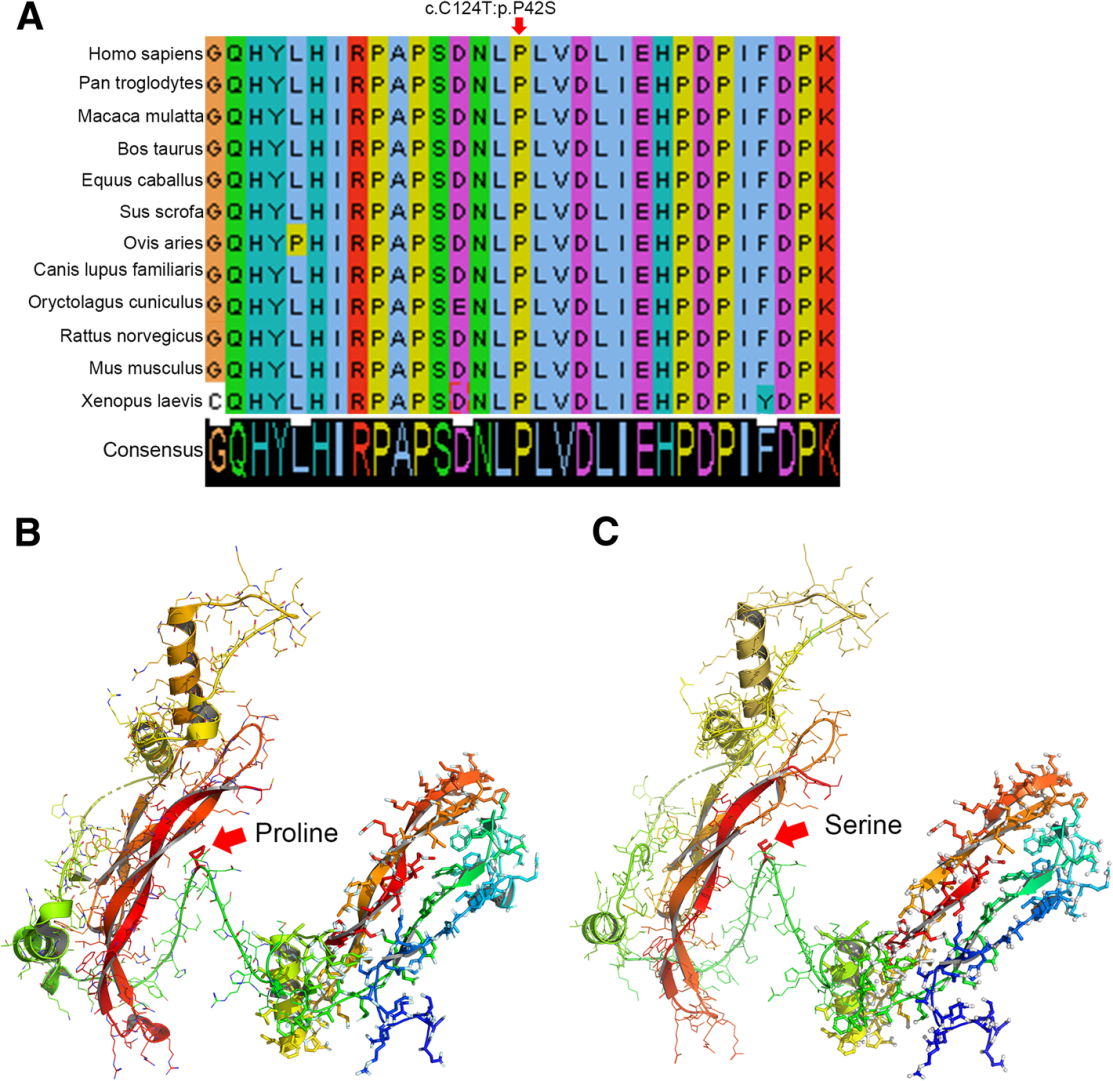
Three-dimensional model of noggin-BMP7 tetramer. **a** Alignment of noggin amino-acid sequences from multiple species. The affected amino-acid site affected by our novel mutation is highly conserved during evolution. **b** Wild-type 3D model of the noggin dimer; BMP7 dimer is on the right. The red arrow indicates position 42 of noggin. **c** Mutation 3D model. On the left is the noggin dimer with c.124C > T, p.(Pro42Ser) mutation; BMP7 dimer is on the right. The red arrow indicates position 42 of noggin

The c.124C > T, p.(Pro42Ser) mutation is predicted to cause a proline-to-serine change at the 42nd amino acid. We aligned noggin amino-acid sequences across multiple species and found that proline at the 42nd position (Pro42) is highly evolutionarily conserved in different species (Fig. 4a). Therefore, Pro42 probably plays a crucial role in maintaining normal noggin function.

*NOG* mutation could decrease noggin binding capacity with bone morphogenetic proteins (BMPs) and GDFs [20]. Through modeling, we measured the interaction capacity between BMP7 and noggin. We found that the mutation site is located in an important turning point of the noggin binding chain (Fig. 4b). Proline is hydrophobic and has a five-membered cyclic side chain, while serine is hydrophilic and acyclic. This variation may change the structure of the binding chain, which in turn affects the combination of noggin and other signals, such as BMPs and GDFs.

## Discussion

Patients in the studied four-generation non-consanguineous family exhibited PIP-joint fusion and talonavicular synostosis without conductive hearing loss, consistent with SYM1. Whole genome sequencing identified a novel missense mutation c.124C > T, p.(Pro42Ser) of *NOG* in these patients, suggesting that this mutation may be the main cause of these patients.

The *NOG* gene (~ 1892 bp) is located on chromosome 17q22 and has only one exon. The gene product is noggin, a 232-amino-acid protein with a molecular mass of 25.774 kDa. The Human Gene Mutation Database has records of multiple *N*OG mutations associated with several disease, including SYM1 [2, 21, 22], brachydactyly type B2 (BDB2) [3, 7, 23], multiple synostoses syndrome 1 (SYNS1, [7, 13]) stapes ankylosis with broad thumbs and toes (SABTT) [3, 22], and tarsal–carpal coalition syndrome (TCC) [7, 24, 25]. Additional file 3 summarizes all known *NOG* mutations that cause symphalangism and related symptoms.

Noggin plays an essential role in cartilage morphogenesis and joint formation [14, 26]. Noggin action occurs through competitive binding with BMP receptors. Due to this function, *NOG* mutations are also commonly associated with conductive hearing loss due to auditory-ossicle fusion [2, 3, 5, 21]. However, our novel c.124C > T, p.(Pro42Ser) mutation did not cause conductive hearing loss, consistent with several previously reported mutations: c. 435C > G, p.(Pro42Arg), c.125C > T, p.(Pro42Leu), and c.124 C > A, p.(Pro42Thr) [27–29]. We note that while two elderly patients (II:1 and II:4) exhibited slight hearing loss, they reportedly had normal hearing when young, suggesting an effect of age rather than genetic mutation. Overall, mutations at the 42nd position does not appear to cause conductive hearing loss. Thus, this symptom is probably closely linked to the exact location of a given *NOG* mutation. More studies are necessary to verify this hypothesis.

Although variants at the amino acid residue of Pro42 did not lead to conductive hearing loss, the symptoms of the patients were diverse. Patients carrying c.435C > G, p.(Pro42Arg) mutations had characteristic facial features of symphalangism, including a hemi-cylindrical nose. One patient exhibited additional features, including ankylosis of proximal and middle phalanges of the hands. Detailed anthropometry revealed relative macrocephaly and shortened spines in affected individuals [28]. Patients carrying c.125C > T, p.(Pro42Leu) mutations presented facial dysmorphism; bulbous nasal tip with a flat nasal bridge; mild hypoplasia of distal fingers with short, flat nails; symphalangism of PIP joints; hypoplasia of middle phalanges; and carpal bone fusion. Radiographs of the feet revealed hypoplastic middle phalanges, talonavicular fusion, osseous fusions of cuneiforms with metatarsals, and cuboid bone absence [27]. Patients with c. 124 C > A, p.(Pro42Thr) mutations exhibit multiple synostoses syndrome (MSS), facial dysmorphism, brachydactylic fingers, as well as incomplete syndactyly of the second, third, and fourth web spaces. Fingers and PIP joins did not present flexion creases, leading to absence of movement in the latter. One patient’s right hand exhibited symphalangism of the third and fourth fingers, while her left hand exhibited symphalangism of the fourth and fifth fingers. In addition, she had a coalition of the trapezium and the first metacarpal [29]. Compared with the other three Pro42 mutations, our novel mutation c.124C > T, p.(Pro42Ser) only led to proximal symphalangism in hands and feet, without any facial dysmorphism. The facial dysmorphism was also not observed in the other studies of Chinese patients with NOG mutations [1, 21, 30, 31]. Together, these findings suggested that *NOG* mutations does not cause facial deformity in individuals of Chinese descent, possibly due to differences in their facial features from people in Europe and America.

In this study, we found evidence that the proline-to-serine mutation may affect binding chain structure in noggin. In turn, noggin binding capacity with other signaling proteins, such as BMPs and GDFs, may be affected. This change may exert a smaller effect than other mutations, perhaps partly explaining why c.124C > T, p.(Pro42Ser) caused only proximal symphalangism without other symptoms.

## Conclusions

In summary, we identified a novel heterozygous *NOG* mutation c.124C > T, p.(Pro42Ser) in a four-generation non-consanguineous Chinese family with SYM1. The affected amino acid was highly conserved across species. The proline-to-serine change resulting from the mutation may alter noggin binding-chain structure, thus influencing noggin binding capacity. Overall, our results indicate that c.124C > T, p.(Pro42Ser) mutation is highly pathogenic and may be the main cause of SYM1 in this family. Our findings broaden the *NOG* mutation spectrum associated with SYM1, clarifying the genetic origins of this condition for the benefit of researchers and clinicians.

## Additional files

Additional file 1: Clinical examination results of the patients participating in the study. (DOCX 14 kb)

Additional file 2: The list of all variants in the proband. (XLSX 2155 kb)

Additional file 3: NOG mutations cause symphalangism and related symptoms. (DOCX 44 kb)

## Data Availability

All data generated or analysed during this study are included in this published article and its supplementary information files.

## Abbreviations

BDB2: Brachydactyly type B2
BMPs: Bone morphogenetic proteins
PIP: Proximal interphalangeal joints
SABTT: Stapes ankylosis with broad thumbs and toes
SNVs: Single nucleotide variants
SYM1: Proximal symphalangism
SYNS: Multiple synostosis syndrome
TCC: Tarsal–carpal coalition syndrome
TPBS: Temtamy preaxial brachydactyly syndrome
WES: Whole-exome sequencing
