# Supplementary material for: Novel NOG (p.P42S) mutation causes proximal symphalangism in a four-generation Chinese family
Source: BMC Med Genet. 2019 Aug 1;20:133. doi: 10.1186/s12881-019-0864-1 (PMC6670124; doi:10.1186/s12881-019-0864-1)
Supplement: Supplementary file 1 — Clinical examination results of the patients participating in the study. (DOCX 14 kb) [file 12881_2019_864_MOESM1_ESM.docx]

**Additional file 1:** **Clinical examination results of the patients participating in the study.**

| Patient | Left hand | Right hand | Left foot | Right foot | Hearing | Thumbs | Face | Hyperopia |
| --- | --- | --- | --- | --- | --- | --- | --- | --- |
| II:1 | pf | pf | df, tf | df, tf | Slight loss | Normal | Normal | Normal |
| II:4 | pf | pf | df, tf | df, tf | Slight loss | Normal | Normal | Normal |
| III:1 | pf | pf | df, tf | df, tf | Normal | Normal | Normal | Normal |
| III:4 | pf | pf | df, tf | df, tf | Normal | Normal | Normal | Normal |
| III:5 | pf | pf | df, tf | df, tf | Normal | Normal | Normal | Normal |
| IV:1 | pf | pf | df, tf | df, tf | Normal | Normal | Normal | Normal |
| IV:3 | pf | pf | df, tf | df, tf | Normal | Normal | Normal | Normal |
| IV:6 | pf | pf | df, tf | df, tf | Normal | Normal | Normal | Normal |

pf, proximal interphalangeal joint fusion; df, distal interphalangeal joint fusion; tf, fusion of the tarsals.
