# Supplementary material for: Novel NOG (p.P42S) mutation causes proximal symphalangism in a four-generation Chinese family
Source: BMC Med Genet. 2019 Aug 1;20:133. doi: 10.1186/s12881-019-0864-1 (PMC6670124; doi:10.1186/s12881-019-0864-1)
Supplement: Supplementary file 3 — NOG mutations cause symphalangism and related symptoms. (DOCX 44 kb) [file 12881_2019_864_MOESM3_ESM.docx]

**Additional file 3:** **NOG mutations cause symphalangism and related symptoms.**

| Mutations | phenotypes | References |
| --- | --- | --- |
| c.915C>G(P35R); c.1376G>T (G189C); c.1460T>G (W217G); c.1470T>A (I220N); c.1470-1471TC>AT(I220N); c.1475T>G (Y222D); c.1476A>G (Y222C); c.1479C>T (P223L) | Failure of joint formation at the proximal interphalangeal (PIP) joints of the hands | (Gong et al. 1999; Marcelino et al. 2001) |
| g.551G>A (C184Y); g.386T>A (L129X); g.58delC (frameshift) | Symphalangism for g.551G>A (C184Y) and g.386T>A (L129X) and multiple synostosis syndrome for g.58delC | (Takahashi et al. 2001) |
| c. 435C>G (P42R); c. 568A>G (M190V) | facial features of symphalangism, including a hemi-cylindrical nose, ankylosis of the proximal and middle phalanges of the hands | (Oxley et al. 2008) |
| c.328C>T (Q110X); c.252-253insC; | congenital stapes ankylosis syndrome: hyperopia, a hemicylindrical nose, broad thumbs and great toes, and other minor skeletal anomalies with conductive hearing loss. | (Brown et al. 2002) |
| c.914C>T (P35R) | bilateral symphalangism | (Mangino et al. 2002) |
| c.142 G>A (E48K) | Premature ovarian failure in a female with proximal symphalangism | (Kosaki et al. 2004) |
| c.1426G>C (W205C); | progressive multiple joint fusions, hyperopia, early-onset conductive deafness, and a typical facies.syndrome. | (Declau et al. 2005) |
| c.615G>C (W205C) | Proximal symphalangism, conductive hearing loss due to stapes fixation and a distinctive facies. | (van den Ende et al. 2005) |
| c130–131insGG (V44fs); c.608T>C (L203P) | Teunissen-Cremers syndrome | (Weekamp et al. 2005) |
| c.103C>G (P35A); c.103C>T (P35S); c.106G>C (A36P); c.142G>A (E48K); c.559 C>T (P187S) | terminal deficiency of fingers and toes | (Lehmann et al. 2007) |
| c.103C>T (P35S); | Teunissen–Cremers syndrome and multiple joint-fusion syndromes | (Hirshoren et al. 2008) |
| c.391C>T (p.Gln131X);  c.304del (p.Ala102fs | Proximal symphalangism, hyperopia, conductive hearing impairment | (Thomeer et al. 2011) |
| c.137T>C (L46P) | Stapes ankylosis, proximal interphalangeal joint fusion, skeletal anomalies and conductive hearing loss. | (Athanasakis et al. 2012) |
| c. 551 G*>*T (C184F); c. 463 T*>*A (C155F); c. C215X | congenital stapes ankylosis with symphalangism, but not in otosclerosis | (Usami et al. 2012) |
| c.452C>A (p.Ser151*); c.261 262ins(G) (p.Pro88Alafs*94); c.125C>T (P42L) | Facial dysmorphism, progressive fusion of multiple joints. | (Lee et al. 2014) |
| c. 124 C>A (p.P42T) | Proximal symphalangism of the fingers and/or toes often associated with fusion of carpal and tarsal bones. | (Aydin et al. 2013) |
| c.499C>T (R167C) | Fusions of the bilateral proximal interphalangeal joints without conductive hearing loss. | (Liu et al. 2014) |
| c.406C>T; (R136C) | Proximal symphalangism and conductive hearing loss | (Masuda et al. 2014) |
| c.559C>G (p.P178A) and c.682T>A (p.C228S) | Proximal symphalangism with hearing loss | (Ganaha et al. 2015) |
| c.682 T> G p.(C228G) | Stapes ankylosis with broad thumbs and toes with conductive hearing loss | (Ishino, Takeno, and Hirakawa 2015) |
| c.450G>C p.(W150C) | proximal symphalangism and conductive hearing | (Pang, Wang, et al. 2015) |
| c.328C>T (Q110X); | Otosclerosis and bilateral hearing loss | (Quesnel et al. 2015) |
| c.689 G>A (C230Y) | Facioaudiosymphalangism syndrome without hearing loss | (Bayat et al. 2016) |
| c.397A>T; p.K133* | dactylosymphysis and conductive hearing loss due to congenital stapes ankylosis. | (Takano et al. 2016) |
| c.611G>A p.(R204Q) | Fusion of the carpals, tarsals, and phalanges with short metacarpals, brachydactyly and humeroradial fusion | (Das Bhowmik, Salem Ramakumaran, and Dalal 2018) |
| c.688_699del, p.(Cys230_Cys232delins11) | Facio-audio-symphalangism syndrome with stapes fixation, syndactyly and symphalangism. | (Westergaard-Nielsen et al. 2018) |
| c.163G > T; p.(Asp55Tyr) | Fusion of proximal interphalangeal joints in the hands and feet. | (Xiong et al. 2019) |
